# Valorization of Onion By-Products Bioactive Compounds by Spray Drying Encapsulation Technique

**DOI:** 10.3390/foods14030425

**Published:** 2025-01-28

**Authors:** Federica Flamminii, Giulia D’Alessio, Marco Chiarini, Alessandro Di Michele, Alessandra De Bruno, Dino Mastrocola, Carla Daniela Di Mattia

**Affiliations:** 1 Department for the Promotion of Human Sciences and Quality of Life, San Raffaele Roma Open University, Via di Val Cannuta 247, 00166 Rome, Italy; alessandra.debruno@uniroma5.it; 2 Department of BioScience and Technology for Food, Agriculture and Environment, University of Teramo, Via R. Balzarini, 1, 64100 Teramo, Italy; gdalessio@unite.it (G.D.); mchiarini@unite.it (M.C.); dmastrocola@unite.it (D.M.); 3 Department of Physics and Geology, University of Perugia, Via Pascoli, 06123 Perugia, Italy; alessandro.dimichele@unipg.it

**Keywords:** onion by-products, flavonoids, encapsulation, spray-drying, storage stability

## Abstract

The increasing interest in sustainability has driven research into the utilization of food by-products. Onion by-products, rich in bioactive compounds, represent a valuable resource for developing functional ingredients; however, they are prone to degradation due to environmental factors such as light, heat, and oxygen, leading to reduced efficacy and increased spoilage. Microencapsulation represents an effective approach to meet important goals in the formulation of food products such as the protection against degradation or the control of interactions with other ingredients that may modify and impair their functionality. This study explores the microencapsulation of flavonoid-rich onion by-product extract through spray drying, employing various wall materials (maltodextrin and a mixture of maltodextrin/trehalose and maltodextrin/trehalose/inulin) and their effect on the chemical and physical properties of the powders such as encapsulation efficiency, total flavonoids content, moisture content, water activity, bulk density, and bulk tapped density. The storage stability was further evaluated. This research supports waste reduction and suggests strategies for developing functional ingredients with extended shelf life and controlled release properties.

## 1. Introduction

Onion (*Allium cepa* L.) is the most widely harvested fresh vegetable in the European Union, with annual production exceeding 6 million tons [1]. Its processing generates substantial quantities of non-edible waste, including onion skins, outer fleshy scales, roots, and bulb tops, primarily resulting from mechanical peeling or discarded due to deformation or damage [2]. Treated as waste, onion by-products are instead rich in functional ingredients with proved health-promoting properties, such as antimicrobial, anti-inflammatory, and antioxidant effects [3,4,5,6].

The polyphenolic profile of onion is dominated by flavonoids, with quercetin and its glucoside derivatives (e.g., quercetin-4′-glucoside and quercetin-3,4′-diglucoside) being the most represented [7] and with well know documented anti-inflammatory, antioxidant, and anticancer properties [8,9,10]. Notably, flavonoid concentrations are highest in onion skins, decreasing from the outer to the inner scales and from the top to the bulb’s base [11]. In contrast, anthocyanins show minimal variation across onion layers [12]. Other polyphenols, such as naringenin, catechins, caffeic acid, and ferulic acid, have also demonstrated beneficial effects, including α-amylase inhibition, which can help regulate blood sugar levels [13,14].

The recovery of phytochemicals from onion waste, particularly flavonoids and anthocyanins, can be considered a crucial step for both reducing the environmental impact of food waste disposal and producing high-value bioactive ingredients for industrial exploitation in the food, pharmaceutical, and cosmetic industries [15]. Onion waste/skin powder or extract was used in bakery products [16,17,18], meat [19], fish [20] preparation, and pasta [21].

Despite their potential, phenolic-rich extracts face challenges such as chemical and physical instability under typical processing and storage conditions (e.g., temperature, oxygen, and light). Microencapsulation offers a promising solution to these drawbacks, enhancing polyphenol stability, bioavailability, and shelf life while masking unpleasant tastes [22]. Among encapsulation technologies, spray-drying is particularly suitable for food applications due to its simplicity, cost-effectiveness, and scalability [23]. Encapsulated onion extracts can serve as stable functional food ingredients, reducing reliance on synthetic additives and aligning with consumer preferences for natural products. Microencapsulated onion skin flavonoid-rich extract was used in cakes, crackers, nachos, and salad dressing [24,25,26,27]. This approach also enables controlled release and improved bioaccessibility, fostering sustainable and resilient food systems [28,29].

However, for a successful spray-drying encapsulation, besides the proper technological parameters to be selected, the choice of the wall material plays a critical role not just for the retention of bioactive compounds in sè but also for their overall stability, functionality, and final exploitation [30]. In the present work, trehalose (TR) and inulin (INU) were studied, and used in combination with maltodextrins (MD); the last are among the most used carriers in spray-drying due to their excellent properties as wall materials. Indeed, they are cost-effective, present a neutral taste, have good solubility, and, more importantly, if the appropriate molecular weight is chosen, their glass transition temperatures (*Tg*) are sufficiently high to provide the formation of a protective matrix with limited hygroscopicity, preventing further degradation during the storage of the powders. Trehalose (α-d-glucopyranosyl-(1→1)-α-d-glucopyranoside), a disaccharide known for its ability to stabilize proteins in low-moisture environments, has been widely used in food encapsulation applications, thanks to its high *Tg*, low hygroscopicity and its ability to form amorphous solids [31]. However, it is rarely used alone as lower encapsulation efficiency was observed when compared with other carrier mixtures. Inulin is a prebiotic fiber composed of fructose polymers which is gaining more attention as an encapsulating agent especially due to its health-promoting properties as a functional ingredient and its high thermal stability. Combinations of various encapsulant polymers can be strategically employed to customize the release of encapsulated bioactive substances. For instance, maltodextrin and inulin have been utilized in the encapsulation of Cactus pear (*Opuntia ficus-indica*) extract [32,33]. These polymers have also been applied to produce microcapsules containing anthocyanins, such as those derived from chokeberry [34], and hibiscus (*Hibiscus sabdariffa* L.) calyces [35]. The co-microencapsulation of flavonoids extracted from yellow onion skins with *Lactobacillus casei* has been successfully achieved using inulin and maltodextrin as encapsulating agents [36]. Similarly, microparticles containing inulin, hi-maize, and trehalose were produced through spray drying to encapsulate *Lactobacillus acidophilus* La-5 [37]. Inulin and maltodextrin have also been used as encapsulating matrices for spray-dried ginger and rosemary essential oils [38,39]; recently, their combination in spray-dried aqueous South American Sapote (*Matisia cordata*) extracts, allowed the production of thermally stable microparticles with spherical morphology and with an improved retention of volatile and phenolic compounds [40]. The impact of spray-drying nanoemulsions with corn oil and various wall materials, including maltodextrin, trehalose, or a combination of both, was also explored [41]. Furthermore, research has been conducted to enhance the stability of roselle anthocyanins through spray-drying employing either a single matrix (maltodextrin) or a binary matrix (maltodextrin-trehalose) [42]. The spray-drying technique was also successfully applied for the encapsulation of phenolic compounds extracted from food wastes such as olive pomace [43,44], blueberry [45], red chicory and red cabbage [46], and orange peel [47].

The present study thus aimed to investigate the use of such different carriers in combination for the spray-drying encapsulation of phenolic-rich extracts obtained from the wastes of “Rossa Di Tropea” onion, a characteristic cultivar planted in the Calabria region (Italy). The effects of these carriers on the chemical, physicochemical, and physical properties of the resulting microencapsulated powders were evaluated. Furthermore, the chemical and functional stability of both non-encapsulated extracts and microparticles during storage under different temperature conditions was also investigated.

## 2. Materials and Methods

### 2.1. Materials

Trehalose was obtained from Adea S.r.l. (Busto Arsizio, Italy). Maltodextrin, GLUCIDEX^®^ 17 with Dextrose Equivalent (DE) value of 17, average Molecular Weight 1059 g/mol, easily soluble and easy to use for spray-drying applications, was kindly donated by Roquette (Lestrem, France). Inulin derived from chicory root (Fibruline^TM^ XL, degree polymerization ≤ 20) was donated by VICTA Food SRL (Mogliano Veneto, Italy) on behalf of Cosucra Groupe Warcoing S.A. (Warcoing, Belgium). The onion by-product phenolic extract (MCE 25) used in this study was kindly provided by the Agricultural Department of the Mediterranean University of Reggio Calabria (Reggio Calabria, Italy). The MCE 25 extract was produced by a conventional solid-liquid extraction throughout the maceration of the onion by-products in 50% (*v*/*v*) ethanol for 120 min at 25 °C, which resulted in the best combination tested [7]. The chemical-functional composition of MCE 25 is reported in Table 1 as indicated by the provider. All the reagents used in this study were of analytical grade.

### 2.2. Spray Drying

In this study maltodextrin (MD), trehalose (TR) and inulin (IN) were used as wall material and mixed in ultra-pure water in different combinations to form three distinct systems: MD/TR, MD/IN, MD/TR/IN in order to achieve a total solid content of 30% (*w*/*w*). Onion extracts enriched powder was prepared by adding 10 mL of MCE 25 extract to each wall material solution. When heating was necessary for the solubilization of wall material, solutions were stirred at 30 °C and 70 °C for MD and IN, respectively, and the amount of evaporated water was added. The heated solutions were cooled at 25 °C and mixed with the extract, sonicated, and fed to a Mini Spray Dryer B-290 (Büchi technology, Flawil, Switzerland) equipped with a 0.7 mm fluid nozzle. The following operating parameters were chosen for the spray drying process: 150 °C as air inlet temperature, 85 ± 10 °C as air outlet temperature, and a feed rate of 30%. Control or blank systems were labeled as MD/TRb, MD/INb, MD/TR/INb, while the enriched microparticles were MD/TR, MD/IN, MD/TR/IN.

### 2.3. Moisture Content and Water Activity

Water activity (a_w_) was measured at 25 °C using a dew point hygrometer Aqua Lab 4TE (Aqua Lab, Decagon Devices Inc., Pullman, WA, USA). A sample dish was carefully filled halfway with microcapsules powder and results were recorded after the equilibrium was reached. Moisture content (MC) was measured gravimetrically by oven drying at 105 °C overnight using 0.5 g of microcapsule powder. The measurements were performed in triplicate and results have been expressed as a percentage of moisture content according to the following formula:

MC (%) = ((*mP*_0_ − *mDP*)/*mP*_0_) × 100

where *mP*_0_ is the mass of powder before drying (in g), while *mDP* is the mass of powder immediately after drying (in g).

### 2.4. Bulk Density, Bulk Tapped Density, Flowability and Cohesiveness

The bulk density (ρb) and bulk tapped density (ρt) of encapsulated onion extract powders were determined according to the method described in the literature [38]. A pre-weighed graduated cylinder of known volume (2 mL) was gradually filled with the powder. After weighing the filled cylinder, the bulk density was calculated by the mass/volume ratio (g/mL). The tapped density was obtained by tapping manually, 5 times, a 5 mL graduated cylinder with approximately 300 mg of powder until an insignificant change in volume between successive measurements was verified. Given the mass (m) and the apparent tapped volume (v) of the powder, the %t was expressed as m/v (g/cm^3^). The flowability and cohesiveness of the powder were determined in terms of Carr index (CI) and Hausner ratio (HR) using the bulk and tapped density, according to the following equations [48]:

CI (%) = [[(qtapped) − (ρbulk)]/(qtapped)] × 100

*H* = (qtapped)/(ρbulk)

The classification of the powders, based on CI and HR values is presented in Table 2.

### 2.5. Water Solubility Index, Water Absorption Index

Water solubility index (WSI) and water absorption index (WAI) were determined according to Paini and co-authors [49] with slight modification. A mass of 167 mg of powder was dispersed in 2 mL of distilled water using a magnetic stirrer for 5 min at ambient temperature. The solution was then centrifuged (Neya 16R, REMI, Vasai, India) at 10,000 rpm for 10 min. The supernatant was transferred to a pre-weighed aluminum dish and oven-dried at 70 °C overnight, while the residue pellet was weighed.

WSI and WAI were calculated according to the following equations:

WSI = *DWsup*/*DWpart* × 100

WAI = *PW*/*DWpart*

where *DWsup* is the dry weight of the supernatant, *DWpart* is the initial weight of microparticles (dry basis) and *PW* is the weight of pellet after centrifugation.

### 2.6. Phenol Extraction from Microparticles

One hundred milligrams of powder microparticles were dispersed in 1 mL of EtOH: Acetic acid: H_2_O in the ratio 50:8:42 (*v*/*v*/*v*), then vortexed for 1 min and extracted twice in an ultrasounds bath (LAB SONIC LBS 1, Falc Instruments, Treviglio, Bergamo, Italy) for forty minutes at 25 ± 3 °C. Samples were then centrifuged at 6000 rpm for 15 min (Neya 16R, REMI, Vasai, India) and the supernatant was collected for the evaluation of the total phenolic (TPC) and flavonoids (TF) content, as well as for the determination of the antioxidant properties by ABTS and FRAP assays. Freeze-dried MCE 25 extract (about 20 mg) was resuspended in 500 mL of 50% (*v*/*v*) of ethanol and used for the same evaluation as previously indicated (TPC, TF, ABTS, FRAP).

### 2.7. Total Phenolic Content (TPC)

TPC was evaluated by using the Folin-Ciocalteau reagent [50]. In brief 120 μL of sample as it is or diluted (1:10 *v*/*v*) was added to 600 μL of 10% (*v*/*v*) Folin-Ciocalteu solution (Sigma-Aldrich, Darmstadt, Germany) in deionized water. The mixture was kept in the dark at room temperature for 2 min, then 960 μL of Na_2_CO_3_ (7.5% *w*/*v*) were added and incubated at 50 °C for 5 min. The total polyphenols content was determined at 760 nm using a spectrophotometer (Lambda Bio 20, Perkin Elmer, Boston, MA, USA). Results were expressed as mg GAE g^−1^ of dry matter or powder, in the case of not encapsulated extract or microparticles, respectively.

### 2.8. Total Flavonoids (TF)

The analysis of total flavonoid content was assessed according to Robert et al. [51] with slight modifications. Fifty hundred microliters of sample were mixed with 500 µL of AlCl_3_ 6H_2_O (2% *w*/*v*), and left to react in the dark for 10 min and at ambient temperature. At the end of the reaction, samples were centrifuged for 3 min at 10,000 rpm (Neya 16R, REMI, Vasai, India). The supernatant was carefully recovered, and placed in the cuvette, and absorbance was read at 430 nm using a spectrophotometer (Lambda Bio 20, Perkin Elmer, Boston, MA, USA). Control samples were used as blank. Results were expressed as mg Quercetin Equivalent (QE) g^−1^ of dry matter or powder using an external calibration curve prepared with Quercetin (3–100 mg kg^−1^).

### 2.9. FRAP

Ferric Reducing Antioxidant Power was determined according to the method described in the literature [52], with slight modifications. In brief 200 μL of sample opportunely diluted was mixed with 1.3 mL of FRAP reagent, obtained by mixing acetate buffer (300 mM, pH 3.6), 10 mM TPTZ (2,4,6-tripyridyl-s-triazine) solubilized in HCl 40 mM and FeCl_3_ 20 mM, in the ratio 10:1:1. The solution was vortexed and incubated at 37 °C for 30 min. The absorbance was measured at 593 nm using a spectrophotometer (Lambda Bio 20, Perkin Elmer, Boston, MA, USA) and FeSO_4_·7H_2_O standard solution was used to calibrate the method. Results were expressed as μmol Fe^2++^Eq g^−1^ of dry matter.

### 2.10. ABTS

The antioxidant activity of the samples was evaluated by the ABTS^•+^ (2,2′-azinobis-(3-ethylbenzothiazoline-6-sulfonic acid)) radical cation decolorization assay. An aliquot of 60 μL of sample opportunely diluted, was mixed with 1440 μL of ABTS radical. The bleaching of the sample-radical mixture was evaluated spectrophotometrically at 734 nm using a spectrophotometer (Lambda Bio 20, Perkin Elmer, Boston, MA, USA). The initial and final absorbance, after 7 min, was recorded, and the inhibition rate was calculated and plotted against the concentration. The angular coefficient (m) of the dose-response curve was compared with that of Trolox, used as a standard. Results were expressed as µmol TEAC g^−1^ dry matter.

### 2.11. Encapsulation Efficiency (EE%)

The encapsulation efficiency (EE%) of the onion enriched powders was determined as follows:

$$EE\%=\frac{(mg\;Quercetin/g\;dm)IS}{(mg\;Quercetin/g\;powder)powder}\times 100$$

i.e., the ration of the content of total flavonoids in the initial solution (IS), before spray drying, to the content of total flavonoids in the powder, both expressed on dry matter (dm).

### 2.12. FT-IR Investigation

FT-IR spectroscopy was used to evaluate polyphenols interactions in onion extract-enriched spray-dried microparticles. Dried structuring agents, dried control, and enriched particles were measured without any special preparation. The infrared spectra were acquired on a Perkin Elmer model Spectrum Two FTIR Spectrometer, based on a Universal Attenuated Total Reflectance sensor (UATR-FTIR). A range from 4000 to 400 cm^−1^ was scanned, with a resolution of 4 cm^−1^ and 4 scans. The spectra of each sample were acquired with six replicates. The samples were positioned on top of the ZnSe-Diamond crystal and the “pressure arm” of the instrument was used to apply constant pressure to ensure good contact between the sample and the incident IR beam, thereby minimizing loss of the IR beam.

### 2.13. Scanning Electron Microscopy (SEM) Analysis

The microstructure of the spray-dried microparticles and their surface morphology was assessed by using a field-emission scanning electron microscope (FE-SEM) LEO 1525 (ZEISS, Oberkochen, Germany) [53]. Prior to image acquisition, sample powders were immobilized on stubs and coated with a thin layer of chromium (8 nm). Acceleration potential voltage was maintained at 5 kV.

### 2.14. Storage Stability

MD/INU microparticles, resulting in those with the highest encapsulation efficiency, were selected for the stability test. Two hundred milligrams of powder were accurately weighted, sealed in glass jars, and stored in dark conditions at 20 ± 1 °C and 50 ± 1 °C until 4 months. At each sampling (0, 3, 6, 13, 60, 90, 120 days), TPC, TF, FRAP, and ABTS, following the methods described previously, were assessed on the non-encapsulated freeze-dried extracts and on the enriched microparticles. Three independent aliquots of each sample were prepared for each sampling time (*n* = 42).

### 2.15. Statistical Analysis

All experiments were carried out in triplicate. Results are reported as mean and standard deviation. A one-way analysis of variance (ANOVA) and Tukey’s test were used to establish the significance of differences among the mean values at the 0.05 significance level; data analysis and modeling were carried out by XLSTAT software v2016 (Addinsoft SARL, New York, NY, USA).

## 3. Results

### 3.1. Powders Physical Properties

Spray drying is recognized as an efficient and industrialized method for the production of food powders, from both single powder particle and bulk powder perspective. The morphology and internal structure of individual particles are used to determine the bulk characteristics of the powder. Additionally, stickiness is a key parameter to evaluate the handling properties and storing ability, as it impacts flowability and rehydration in terms of speed and degree, respectively, that are critical to achieving proper powder properties [54].

The physical properties of the control and enriched spray-dried powders are presented in Table 3.

#### 3.1.1. Moisture Content and Water Activity

The moisture content of the control and enriched microparticles (Table 3) appeared higher when inulin was used, mainly in the control system MD/INUb and in MD/TR/INU. The use of polymers such as inulin can lead to the production of powders with higher moisture content, which is a consequence of the higher drying rate of such particles that cause the formation of a crust that hinders the water diffusion and the evaporation process [38]. Further, high DE maltodextrins can increase the moisture content of powders due to their lower molecular weight which implies the presence of shorter chains and more hydrophilic groups [55]. Indeed, as reported in the literature [56], studying the effect of 6, 12, and 21 DE maltodextrins on the moisture content of orange juice powders, the authors verified that increasing water contents were obtained with the increase of maltodextrin dextrose equivalent c. Further, a reduction of DE, from 10 to 6, resulted in a reduction of moisture content due to the lower binder propertied of the latest [57]. The observed moisture content, regardless of the wall material used, resulted lower than values observed by natural extracts obtained from grape skins of “*Barbera*” [58], from olive pomace polyphenols-rich extracts [44], and from blueberry waste extract [45], using maltodextrin and inulin as wall materials, ranging from 3.6 to 11.2%.

Water activity, unlike moisture content, indicates the availability of free water in a food system, which drives chemical and biochemical reactions. Moisture content, on the other hand, measures the total water present. High water activity promotes spoilage and reduces shelf life [59]. The water activity of control microparticles ranges from 0.09 to 0.11, lower than systems enriched with onion extract, which ranges from 0.12 to 0.13. The results appear in line with the study in which the encapsulation of Sea Buckthorn Juice with maltodextrin and inulin matrix was explored, with values ranging from 0.08 to 0.09 [60]. Overall, microencapsulated powders showed a_w_ values lower than 0.15 and moisture content lower than 3% which ensure the reduction of microbial risk, enhance their solubility, improve their overall storage stability, and make them well-suited for industrial applications [61].

#### 3.1.2. Bulk Density, Bulk Tapped Density, Flowability and Cohesiveness

The bulk density of control and encapsulated systems (Table 3) ranges from 0.44 to 0.45 g mL^−1^ and 0.36 to 0.37 g mL^−1^, respectively. Control samples showed higher bulk density than microparticles, regardless of the wall material used for the encapsulation.

The bulk tapped density is an important factor related to the transport, packaging, and marketing of powders; thus, this variable is useful for determining the weight and amount of material that will fit inside a container [62]. A high-density dry product can be stored in a smaller container, compared with a low-density product [63]. In the control samples (Table 3) there are differences among the three systems as the lowest value is presented for the MD/TRb with 0.54 g mL^−1^ while higher values were reported for the systems with inulin, MD/INUb and MD/TR/INUb with 0.58 and 0.61 g mL^−1^, respectively. The same behavior is reported for systems encapsulated with MCE 25 as the lowest value is found in MD/TR with 0.56 g mL^−1^, while for MD/INU and MD/TR/INU, higher values with 0.64 and 0.62 g mL^−1^, respectively.

The results highlight the effect of polymers such as inulin and maltodextrin in increasing the bulk density in agreement with the study of Fernandes and coauthors [38], in which Arabic gum, starch, maltodextrin, and inulin were used as wall materials for the microencapsulation of rosemary essential oil with results ranging from 0.35 g mL^−1^ to 0.49 g mL^−1^. Furthermore, an increase in the dextrose equivalent (DE) of maltodextrin resulted in a higher bulk density. This phenomenon can be explained by the relationship between maltodextrin’s DE and its glass transition temperature (*Tg*). As the DE increases, the *Tg* decreases, leading to a stickier mixture that contributes to the observed rise in bulk density [56].

Flowability, represented by the Carr index (CI), is the relative movement of the particles either among themselves or along the surface of a vessel wall, while cohesiveness, measured by the Hausner Ratio (HR) is an internal property of the powder indicating the internal forces that bind particles together. The lower the Carr index and Hausner ratio, the better the flow, and the less cohesive the powder [64]. The flowability of the encapsulated systems resulted in very limited due to CI% values ranging from 37.74 to 43.20. Conversely, cohesiveness is intermediate for the control system and high for the encapsulated one with values > 1.4. An explanation may rely on the particle size of the powder: at small particle sizes, the large surface area per unit mass of powder increases the contact surface area between powder particles available for cohesive and frictional forces to resist flow; conversely, as observed by Landillon et al. [65], an increase in particle size tends to decrease the cohesion because particle surface area per unit mass decreases, reducing the surface area for inter-particle bonding and interactions, thus decreasing cohesiveness and resulting in increase in flowability; generally, the higher the cohesiveness, the larger the powder collapse observed on tapping [66]. Furthermore, an increase in the moisture content of powders has been found to increase the cohesion between powder particles, resulting in lower flowability. This latest aspect could be associated with the significant differences in water activity instead of moisture content among the systems, as previously discussed. Besides, the surface charge density of the powder, affected by wall the material and phenolic compounds nature (i.e., charge: negative for polyphenols and positive for anthocyanins [67]), could influence the particle-particle interaction and the final performances of the powders [68].

#### 3.1.3. Water Solubility Index, Water Absorption Index

Solubility is an important quality factor influencing the reconstitution behavior of microencapsulated extracts and the release of the core material [61]. Generally, it depends on the surface area and particle size, and the presence of amphipathic substances and their chemical structure [69]. The WAI value (Table 3) depicted the lower values for the system containing trehalose, in both control and enriched samples while the addition of inulin caused an increase the WAI value, especially in the system without trehalose. Beet Root Extract encapsulated with maltodextrin and inulin showed values of 27.09 and 30.68, respectively, highlighting a higher WAI in the maltodextrin system due to its higher solubility when compared to inulin [70].

WSI was high in the trehalose-containing systems, in both control and enriched ones. The addition of small sugars such as trehalose increases the interaction with water improving the solubility. Similarly to the work by Lacerda and co-authors [71], in which inulin and maltodextrin are used as encapsulating agents of jussara pulp microparticles, WSI ranged from 76.8% to 85.0% indicating that all selected microparticles were adequately soluble in water. Inulin presented the lowest value of WSI, likely due to the reduced availability of hydrophilic groups to bind water; conversely, maltodextrin presented the highest values of WSI, linked to its high capacity to absorb water, with average values of 72–83% [72] compared to inulin, whose values are much lower (15–49%) [73]. Such a difference is directly related to the presence of hydrophilic regions in the chemical structure [74].

#### 3.1.4. Powders Microstructure

**Figure 1:**
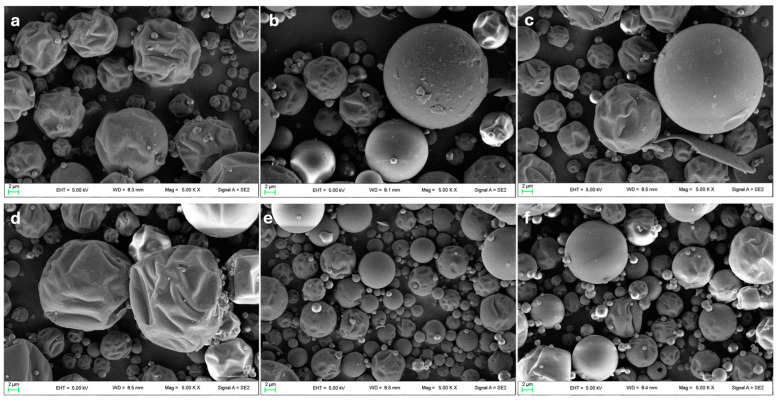
SEM micrographs of reference powders without extract (**a**–**c**), formulated with MD/INUb (**a**), MD/TRb (**b**), and MD/INU/TRb (**c**), and their respective ones encapsulated with onion by-product phenolic extract MD/INU, MD/TR and MD/TR/INU (**d**–**f**).

Figure 1 shows SEM micrographs of MD/INUb, MD/TRb, and MD/TR/INUb (Figure 1a–c, respectively) and encapsulated microparticles MD/INU, MD/TR and MD/TR/INU (Figure 1d–f, respectively). MD/INU microcapsules (Figure 1a,d), regardless of the presence of the extract, exhibited an irregular shape characterized by surface depressions, likely attributable to varying depths of indentation, appearing highly wrinkled. A very similar morphology has also been reported by other authors who used inulin as an encapsulating agent [39,75]. Furthermore, the presence of maltodextrin influences even more the formation of shrunk and concave particles, as well as irregularities on the surface, often reported in the literature [76,77]. The morphology of microparticles obtained with MD/TR combination (Figure 1b) showed a smother surface and a lower percentage of shrunk particles, concavities as well as indentations than microparticles with inulin [37]. Similar results were observed for powders obtained with maltodextrin and trehalose with a smooth surface and homogeneous particles because trehalose is a small carbohydrate, which acts as a plasticizer during the drying process, preventing wrinkling and promoting surface uniformity [78]. Observing MD/INU/TR microparticles (Figure 1c,f), a heterogeneous population is denoted as a result of the mix of the polymers and the relative effects previously described, indeed, particles like those described for the inulin coexist with those obtained with trehalose. The presence of shrunken particles, slight wrinkles, and surface depressions in all the powders, regardless of the presence of the extract, can be attributed to the rapid drying of droplets at high temperatures during the spray-drying process. This rapid drying causes a drastic loss of water vapor, followed by cooling [76,79].

The addition of the extract (Figure 1d–f) did not particularly impact the morphology of microparticles, but rather the formation of smaller particles and even small aggregates, as also observed by Faieta and coauthors [78], with the encapsulation of *Spirulina platensis* extract. Furthermore, the microcapsules do not show wall breaks, demonstrating that the encapsulation has been carried out in the appropriate way [37,76]. The presence of concavities even after the addition of the extract can still be a positive factor, as this structure improves the resistance to diffusion and the ability of the powders to withstand mechanical stresses, thereby providing better protection for the product [76,80,81].

The micrography also revealed particles with diameters ranging from approximately 1 µm to 25 µm. This size range is comparable to microparticles produced using various wall materials combined with inulin, which were used to encapsulate corn oil and exhibited diameters ranging from 0.6 µm to 26 µm [82]. Such size polydispersity is a typical characteristic of particles produced via spray-drying [83]. Furthermore, the fluorescence analysis highlighted the presence and distribution of flavonoid compounds within the matrix, attributed to their natural fluorescence [84].

### 3.2. FT-IR

**Figure 2:**
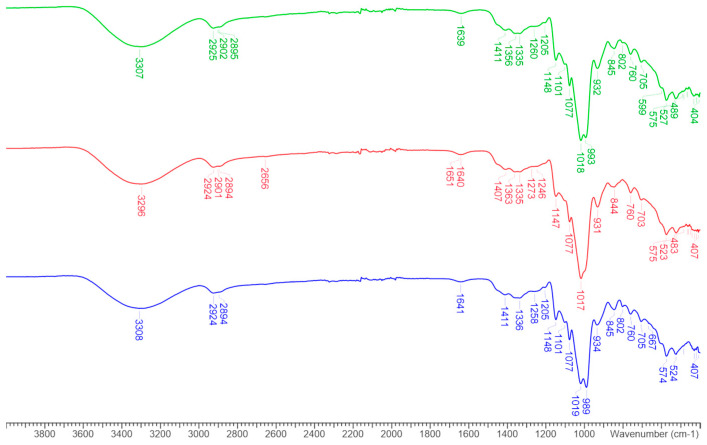
FT-IR spectra of MD/TRb (blue line), MD/INUb (red line) and MD/TR/INUb (green line).

**Figure 3:**
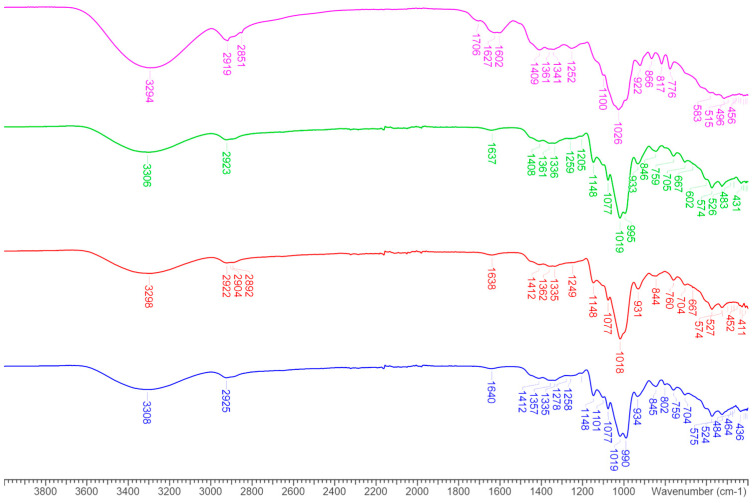
FT-IR spectra of MD/TR (blue line), MD/INU (red line), MD/TR/INU (green line) and pure onion extract (magenta line).

FT-IR spectroscopy was used as a powerful tool for chemical structure elucidation and qualitative compositional analysis and applied for the characterization of the spray-dried microparticles. FT-IR spectra of pure extract, blank and incapsulated microparticles are reported in Figure 2 and Figure 3. No differences were denoted between blank and extract enriched microparticles.

Briefly, all the saccharide spectra (Figure 2) showed a strong and wide O-H stretching peak in the 3600–3100 cm^−1^ range, C-H stretching at 2900 cm^−1^, C-H scissor and bending at 1450–1290 cm^−1^, stretching of alcoholic C-O at 1258 cm^−1^, C-O-C and C-O-H bending at 1100–1060 cm^−1^ and an absorption peak at 932 cm^−1^. Bands in the range 1200–800 cm^−1^ are characteristic of the polysaccharide backbone and comprise C–O stretching, CH_2_ twisting, C–O–H bending, and C–O–C bond stretching vibrations of the polysaccharide chains [85]. The spectrum of onion by-product extract (Figure 3, magenta line) showed the characteristic flavonoid compounds bands in the range 3400–3100 cm^−1^, characteristic for hydroxyl groups and intermolecular hydrogen bonds and with maximum around 3000–2900 cm^−1^ for stretching of C–H, the region 1700–1600 cm^−1^ originating from carbonyl group and the adsorption at 1602 cm^−1^ was assigned to C=C–C aromatic stretching groups. The stretching of ring C–C appears in the region of 1634–1300 cm^−1^ and of the C–O bond at 1368–1157 cm^−1^ and 1031–1023 cm^−1^, C–H bending in plane in the range of 1300–1000 cm^− 1^ and out of plane 700–500 cm^−1^ [86].

The FT-IR absorption spectra for the encapsulated system containing the extract were dominated by polysaccharide chain signals (Figure 3) and were indistinguishable from that of the spray-dried polysaccharide (Figure 2). The lack of formation of new bonds and the absence of signals due to the onion by-product extract is explained by the low concentration of the extract in the spray-dried powders. This result is confirmed by previous studies [87,88], in fact, when the core material is encapsulated inside the particles, the band is overshadowed by that of the wall material, due to the reduction in the concentration of the core material contained inside the particles [76,77]. On the contrary, if the material to be encapsulated is not inside the particles, then its signal will appear in the spectra of the powders.

### 3.3. Total Flavonoids Content and Encapsulation Efficiency

Processing of food, especially thermal processing, often leads to nutrient loss in food products, which makes it an urgent challenge for the food industry to preserve the functional components during processing [89]. Therefore, ensuring the retention of bioactive compounds during processing, including spray-drying, is a key area of interest.

Red onion skins are the richest source of total flavonoids, especially anthocyanins, compared to other onion varieties. The extract used in this study is reported to have a notably high total anthocyanin content, accounting for approximately 0.47 mg C-3-GLUC g^−1^ d.w., applying the conventional extraction method [7].

Feed speed rate and air inlet temperature have been reported as important variables in the spray drying process [90]. In this study, standardized conditions based on an inlet temperature of 150 °C and a feed rate of 30% were used for the three systems prepared as MD/TR, MD/INU, and MD/TR/INU. Considering that the initial TF content of the three feed solutions before spray-drying was about 0.23 mg QE g^−1^ dm, a slight decrease was observed for all the systems after the process (Table 4), reflecting an encapsulation efficiency ranging from 75% to 89%. Results highlighted the higher TF content in MD/INU system (0.22 mg QE g^−1^), followed by the ternary mixture of MD/T/INU and MD/T with 0.19 and 0.17 mg QE g^−1^, respectively.

Cranberry polyphenols are reported to be relatively stable upon thermal processing; indeed, cranberries are a rich source of proanthocyanidins and flavonols, and both were proven to show good thermal stability [91]. Additionally, chemical conformational changes in the phenolic compounds, which eventually occurred during spray drying, may also have enhanced their solubility and extractability in aqueous media in the Folin-Ciocalteau assay, leading to an increase in TPC [92].

The total flavonoid content of the enriched microparticles and their encapsulation efficiency are reported in Table 4. Encapsulation efficiency values exceeded 70%, with the highest efficiencies observed in systems where inulin was used as the wall material, achieving 88% and 89% for MD/TR/IN and MD/IN, respectively. In contrast, the system with only trehalose (MD/TR) showed an encapsulation efficiency of 75%. The slight variation in encapsulation efficiency may be attributed to the different wall materials used, which influence the structure of the microparticles and the interactions between the polymers and onion bioactive compounds. These findings are consistent with those reported by Robert et al. [93], who encapsulated gallic acid (GA) using wall materials like inulin and starch, the latter having similar properties to maltodextrins. Their results indicated that GA encapsulation efficiency was higher for inulin (83%) compared to starch (47%), highlighting the impact of the wall material on the GA-polymer interaction. Although both starch and inulin are polysaccharides, they differ in their structural features. Starch is a glucopyranose with both linear and branched regions [94], whereas inulin is a mainly linear fructo-oligosaccharide (FOS) with slight branching [95]. Furthermore, maltodextrins are known to interact with polyphenols to form complexes, which can enhance their stability [96]. Similar findings were reported by Ding et al. [97], who compared seven carbohydrates as wall materials for microencapsulating lutein powders. Their results showed lower encapsulation efficiencies (56.1% to 61.2%) for maltodextrin and trehalose systems, while inulin-based systems yielded higher encapsulation efficiencies (75%).

### 3.4. Storage Stability

**Figure 4:**
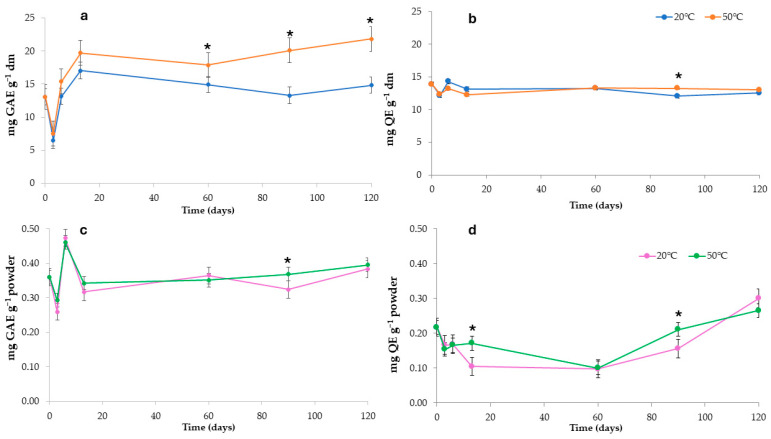
Evolution of TPC (mg GAE g^−1^) and TF (mg QE g^−1^) content during storage of not-encapsulated extract (**a**,**b**) and encapsulated microparticles extract (**c**,**d**). Asterisks mean significant difference (*p* < 0.05) between samples stored at the same time but under different temperatures.

**Figure 5:**
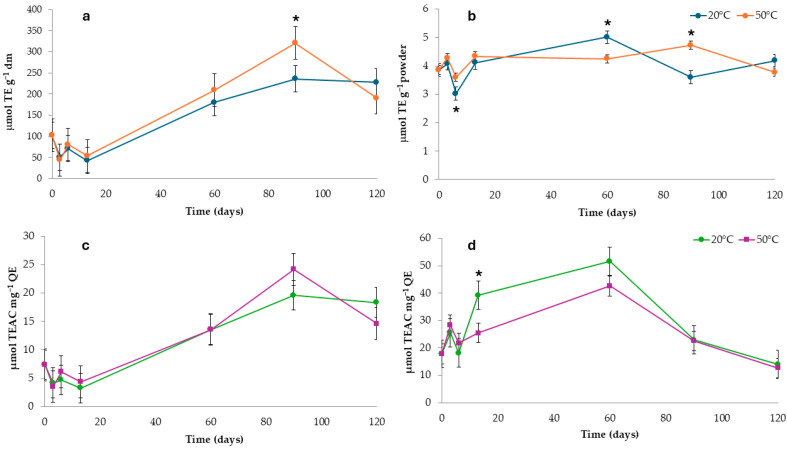
Antiradical activity (μmol TE g^−1^) of onion by-product extract (**a**), encapsulated extract (**b**), and normalized values for total flavonoids content for the onion by-product extract (**c**) and encapsulated extract (**d**). Asterisks mean significant difference (*p* < 0.05) between samples stored at the same time but under different temperatures.

**Figure 6:**
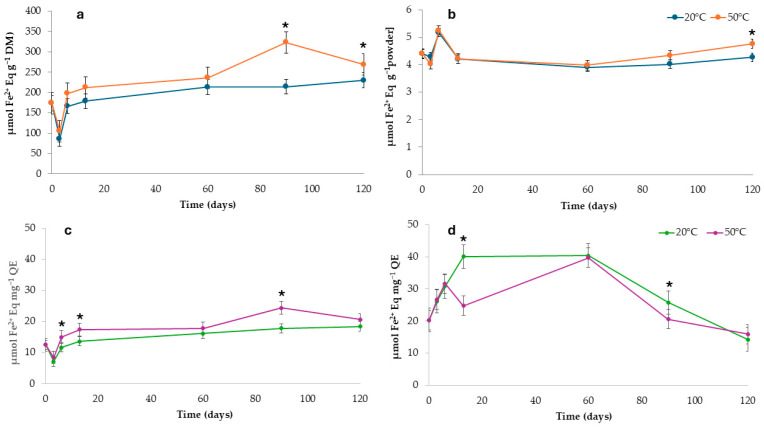
Antiradical activity (μmol Fe^2+^ Eq g^−1^) of onion by-product extract (**a**), encapsulated extract (**b**), and normalized values for total flavonoids content for the onion by-product extract (**c**) and the encapsulated extract (**d**). Asterisks mean significant difference (*p* < 0.05) between samples stored at the same time but under different temperatures.

Microparticles’ chemical stability and antioxidant properties were evaluated considering the system with the highest EE%. To this aim, the variation of TPC, TFC, ABTS, and FRAP in the spray-dried MD/INU powder samples, stored at 20 °C and 50 °C for 120 days, were assessed and presented in Figure 4, Figure 5 and Figure 6. The two storage temperatures were chosen to assess particles and extract stability upon ambient temperature and thermal abuse conditions. In order to get a powder, the non-encapsulated onion extract was freeze-dried and stored under the same conditions.

The total phenolic content (TPC) of the non-encapsulated onion by-product extract (Figure 4a) initially decreased at t2, followed by a gradual increase and stabilization during storage. Samples stored at 50 °C exhibited higher TPC values compared to those stored at 20 °C, with a final variation of approximately 40% and 11%, respectively. As reported in the literature, elevated temperatures can increase free phenolic content by hydrolyzing conjugated polyphenols, such as flavonol glycosides, at the glycosidic bonds between phenolics and sugars. This process produces phenolic aglycones, enhancing their reactivity with the Folin-Ciocalteu reagent [33].

The TPC values of the microparticles (Figure 4c) remained relatively stable up to t90, with values of 0.32 ± 0.02 and 0.37 ± 0.01 mg GAE g^−1^ for storage at 20 °C and 50 °C, respectively. No substantial differences were observed between the two storage temperatures, showing a final variation of approximately 6% (20 °C) and 9% (50 °C) likely due to the protective effect of the wall material in preventing the degradation of bioactive compounds during storage. Chandra et al. [98] suggested that the interaction between the flavylium cation form of anthocyanins and maltodextrin could retard their transformation into less stable forms, thereby enhancing their stability. For example, polysaccharides like maltodextrin have been reported to interact with phenolics to form complexes, which can improve the stability of polyphenols [96]. In contrast, the total flavonoid content (TF) of the non-encapsulated onion extract (Figure 4b) exhibited a fluctuating pattern during the first two weeks, followed by stabilization during the remaining storage period with a final variation of approximately −10% (20 °C) and −6% (50 °C). The TF content of the microparticles (Figure 4d) showed a general decrease during storage, followed by an increase in the final month with final variation at approximately 28% (20 °C) and 18% (50 °C). This trend is likely associated with the hydrolysis of conjugated polyphenols during storage [51]. Notably, however, the decrease was more pronounced at 20 °C than at 50 °C, particularly at t13, with values of 0.11 ± 0.00 mg QE g^−1^ and 0.17 ± 0.01 mg QE g^−1^, respectively.

Typically, the TPC in spray-dried powders is expected to decrease during storage due to decomposition [80]. However, in this study, all phenolic compounds under investigation, including both simple phenolics and flavonoids, showed varying levels of increase during storage. A similar phenomenon was observed in cranberry powders, in spray-dried blueberry pomace extract after 40 days of storage, and in encapsulated grape pomace extract [45,99,100,101]. These studies concluded that TPC and antioxidant capacity may be preserved or even enhanced due to the release of free phenolic compounds during storage, likely resulting from the hydrolysis of conjugated polyphenols. The increase in flavonoids observed over time and at higher temperature-storage combinations may be attributed to the conversion of glycosides or bound phenolics into free phenolic derivatives or the formation of complexes through interactions with metals [102].

The antiradical activity of the non-encapsulated onion by-product extract and microparticles is presented in Figure 5. Results were expressed on a dry matter basis for the pure extract and on a powder basis for the microparticles (Figure 5a,b). Additionally, the values were normalized to the total flavonoid content, the predominant phenolic compounds in red onion skins, to better illustrate their influence on the final outcomes (Figure 5c,d).

This normalization approach reduced the impact of concentration differences and minimized the influence of unrelated variables, such as variations in assay sensitivity, sample size inconsistencies, and the effects of the carrier material. By scaling the data, this method enhanced the clarity of the final signal, mitigating potential interference from size discrepancies and other compounds that could obscure the specific contribution of flavonoids during storage analysis [103].

During storage, a noticeable increase in antiradical activity was observed in the lyophilized extract samples, with slightly higher values in those stored at 50 °C compared to 20 °C, at t90, regardless of normalization against TF values. The behavior of the microparticles, however, was quite different. After an initial two-week period of fluctuating values, TE levels stabilized at approximately 4 µmol TE g^−1^ powder at both storage temperatures with final variation at approximately 8% and −2% for 20 °C and 50 °C, respectively. When examining the normalized values, microparticles stored at 20 °C exhibited higher antiradical activity than those stored at 50 °C between t13 and t60, followed by a decrease that reached a final variation at around −27% and −40% for 20 °C and 50 °C, respectively.

The reducing properties, measured by the FRAP method and calculated in the same manner as for ABTS, are presented in Figure 6. A slight increase in reducing properties was observed in the pure extract samples during storage, with higher values at 50 °C than at 20 °C (Figure 6a), that reached a final variation at around 35% and 25%, respectively, consistent with the trend observed for the antiradical activity assessed by ABST assy. Concerning the microparticles, the normalization against TF content (Figure 6d) revealed higher reducing properties for samples stored at 20 °C compared to 50 °C, at t90, with final variation at around 21% and 2%, respectively, mirroring the pattern previously observed for the antiradical activity.

The normalization of antioxidant activity against TF content revealed distinct storage-dependent behaviors. The non-encapsulated onion extract exhibited higher activity when stored at 50 °C, whereas microparticles showed superior performance at 20 °C.

The high thermo-sensitivity of anthocyanins and their rapid degradation during storage, particularly at elevated temperatures, is well-documented in the literature [104,105,106]. The reduced antioxidant activity observed in microparticles stored at 50 °C can be attributed to the degradation of phenolic compounds, especially anthocyanins, under high-temperature conditions [80]. Conversely, the higher antioxidant activity observed in microparticles stored at 20 °C—peaking at approximately 8 weeks, particularly in the TEAC assay (Figure 5d)—is likely due to the progressive release of phenolic oligomers over time. Zhang et al. [80] reported that room-temperature storage enhanced antioxidant activity in microparticles after about 8 weeks, attributed to the hydrolysis of quercetin glycosides and the resulting formation of protocatechuic acid and quercetin. A similar process likely occurred in the powders analyzed in this study, given the high concentration of phenolic glycosides in the extract, particularly quercetin-3-*O*-glucoside. Over time, the hydrolysis of quercetin-3-*O*-glucoside into quercetin likely contributed to the observed increase in antioxidant activity, as quercetin was already abundant in the extract. Thus, the enhanced antioxidant response can be attributed to the increased release of small phenolic oligomers and the stabilizing effects of room-temperature storage, as also noted by Zhang and colleagues [80]. In contrast, the higher antioxidant activity observed in the non-encapsulated onion extract stored at 50 °C could be attributed to the onset of the Maillard reaction during storage. This reaction may result in the formation of Amadori and Heyns rearrangement products, as well as other intermediate compounds (e.g., α-dicarbonyls), at their initial/intermediate stages, with known antioxidant properties. Notably, amino compounds such as amino acids and reducing sugars may also degrade under heat, producing reactive intermediates with antioxidant properties that can further influence assay responses [80,107].

## 4. Conclusions

This study focused on the characterization of spray-dried encapsulated onion by-product extracts, examining the effects of different wall materials—maltodextrin, trehalose, inulin, and their combinations on the chemical and physical properties of the resulting powders. The findings highlighted the high encapsulation efficiency of the systems evaluated, with the maltodextrin-inulin (MD/INU) combination emerging as the most effective matrix for preserving bioactive compounds. The encapsulated powders exhibited comparable levels of humidity, water activity (aw), and bulk density, with no significant differences observed across the systems. While the MD/INU microparticles showed the lowest water solubility index (WSI), they demonstrated the highest encapsulation efficiency among the wall materials studied. This makes the MD/INU combination the optimal choice for preserving and delivering bioactive compounds in food applications.

Additionally, storage studies revealed that both the free extract and the encapsulated powders retained significant antioxidant activity over time, with storage conditions influencing their behavior. The non-encapsulated extract stored at 50 °C exhibited higher antioxidant activity, likely due to the occurrence of Maillard reactions during storage of the pure extract and the formation of products with antioxidant properties. In contrast, microparticles stored at 20 °C showed better preservation of phenolic compounds and antioxidant activity, attributed to the protective effects of the wall materials and the progressive release of phenolic oligomers. These results underscore the potential of encapsulated onion by-product extracts for maintaining bioactive compound stability and functionality during storage, particularly under optimized conditions.

## Figures and Tables

**Table 1:** The amount of the most representative phenolic compounds of the onion by-product extract used in this study, along with the antioxidant properties.

	MCE 25
Protocatechic acid (mL L^−1^)	21.56 ± 1.48
Rutin (mL L^−1^)	33.29 ± 3.12
Quercetin-3-*O*-glucoside (mL L^−1^)	102.16 ± 9.23
Quercetin (mL L^−1^)	155.28 ± 5.32
Isorhamnetin (mL L^−1^)	6.04 ± 0.07
Isorhamnetin 3-*O*-glucoside (mL L^−1^)	8.88 ± 1.08
TF (mg CE mL^−1^)	1.32 ± 0.05
ABTS (µM TE mL^−1^)	3.45 ± 0.53
DPPH (µM TE mL^−1^)	1.61 ± 0.05

TF: total flavonoids; ABTS: 2,2′-Azino-bis (3-ethylbenzthiazoline-6-sulfonic acid); DPPH: 2,2-diphenyl-1-picrylhydrazyl.

**Table 2:** Carr index (CI) and Hausner ratio (HR) of powders.

CI (%)	Flowability	HR	Cohesiveness
<15	Very good	<1.2	Low
15–20	Good	1.2–1.4	Intermediate
20–35	Fair	>1.4	High
35–45	Bad		
>45	Very bad		

**Table 3:** Physical characterization of control systems and the onion by-products extract encapsulated systems. Mean ± standard deviation of n = 3 repetitions.

	MC%	a_w_	WAI	WSI	Bulk Density g mL^−1^	Bulk Tapped Density g mL^−1^	CI%	HR
MD/TRb	1.3 ± 0.05 ^b^	0.093 ± 0.002 ^b^	0.14 ± 0.03 ^bc^	98.65 ± 0.36 ^a^	0.44 ± 0.01 ^a^	0.54 ± 0.02 ^c^	17.63 ± 1.86 ^d^	1.21 ± 0.03 ^d^
MD/INUb	2.3 ± 0.23 ^a^	0.095 ± 0.007 ^b^	0.30 ± 0.07 ^a^	93.33 ± 0.98 ^b^	0.45 ± 0.02 ^a^	0.58 ± 0.01 ^abc^	21.77 ± 1.87 ^cd^	1.28 ± 0.03 ^cd^
MD/TR/INUb	1.7 ± 0.05 ^ab^	0.106 ± 0.003 ^b^	0.25 ± 0.08 ^abc^	95.68 ± 0.79 ^ab^	0.44 ± 0.01 ^a^	0.61 ± 0.03 ^abc^	27.32 ± 3.00 ^cd^	1.38 ± 0.06 ^c^
MD/TR	1.8 ± 0.08 ^ab^	0.131 ± 0.010 ^a^	0.09 ± 0.02 ^c^	97.82 ± 2.57 ^a^	0.36 ± 0.01 ^b^	0.56 ± 0.03 ^bc^	35.74 ± 2.42 ^b^	1.56 ± 0.06 ^b^
MD/INU	1.8 ± 0.13 ^ab^	0.122 ± 0.005 ^a^	0.28 ± 0.08 ^ab^	92.15 ± 2.62 ^b^	0.36 ± 0.01 ^b^	0.64 ± 0.02 ^a^	43.20 ± 0.95 ^a^	1.76 ± 0.03 ^a^
MD/TR/INU	2.5 ± 0.06 ^a^	0.131 ± 0.005 ^a^	0.13 ± 0.02 ^bc^	95.36 ± 0.75 ^ab^	0.37 ± 0.01 ^b^	0.62 ± 0.01 ^ab^	40.24 ± 1.48 ^ab^	1.67 ± 0.04 ^ab^

Value in column with different letters are statistically different at *p* level < 0.05.

**Table 4:** Total flavonoid content of the initial feed solution, microparticles, and encapsulation efficiency.

	Feed Solution mg QE g^−1^ dm *	Microparticles mg QE g^−1^ Powder	EE%
MD/T	0.23 ± 0.01	0.17 ± 0.01	75 ± 3
MD/INU	0.24 ± 0.03	0.22 ± 0.01	89 ± 10
MD/T/INU	0.22 ± 0.01	0.19 ± 0.01	88 ± 3

* Initial TF content of feed solution with MCE25 extract.

## Data Availability

The original contributions presented in the study are included in the article, further inquiries can be directed to the corresponding authors.

## References

[1] European Union Fruit and Vegetable Production in 2022—Eurostat. https://ec.europa.eu/eurostat/fr/web/products-eurostat-news/-/ddn-20240301-1.

[2] El Mashad H.M., Zhang R., Pan Z. (2019). Onion and Garlic. Integrated Processing Technologies for Food and Agricultural By-Products.

[3] Griffiths G., Trueman L., Crowther T., Thomas B., Smith B. (2002). Onions—A Global Benefit to Health. Phytother. Res..

[4] Celano R., Docimo T., Piccinelli A.L., Gazzerro P., Tucci M., Di Sanzo R., Carabetta S., Campone L., Russo M., Rastrelli L. (2021). Onion Peel: Turning a Food Waste into a Resource. Antioxidants.

[5] Stoica F., Rațu R.N., Veleșcu I.D., Stănciuc N., Râpeanu G. (2023). A Comprehensive Review on Bioactive Compounds, Health Benefits, and Potential Food Applications of Onion (*Allium cepa* L.) Skin Waste. Trends Food Sci. Technol..

[6] Kumar M., Barbhai M.D., Hasan M., Punia S., Dhumal S., Radha, Rais N., Chandran D., Pandiselvam R., Kothakota A. (2022). Onion (*Allium cepa* L.) Peels: A Review on Bioactive Compounds and Biomedical Activities. Biomed. Pharmacother..

[7] Imeneo V., De Bruno A., Piscopo A., Romeo R., Poiana M. (2022). Valorization of ‘Rossa Di Tropea’ Onion Waste through Green Recovery Techniques of Antioxidant Compounds. Sustainability.

[8] Almeida A.F., Borge G.I.A., Piskula M., Tudose A., Tudoreanu L., Valentová K., Williamson G., Santos C.N. (2018). Bioavailability of Quercetin in Humans with a Focus on Interindividual Variation. Compr. Rev. Food Sci. Food Saf..

[9] Lesjak M., Beara I., Simin N., Pintać D., Majkić T., Bekvalac K., Orčić D., Mimica-Dukić N. (2018). Antioxidant and Anti-Inflammatory Activities of Quercetin and Its Derivatives. J. Funct. Foods.

[10] Osojnik Črnivec I.G., Skrt M., Šeremet D., Sterniša M., Farčnik D., Štrumbelj E., Poljanšek A., Cebin N., Pogačnik L., Smole Možina S. (2021). Waste Streams in Onion Production: Bioactive Compounds, Quercetin and Use of Antimicrobial and Antioxidative Properties. Waste Manag..

[11] Pérez-Gregorio R.M., García-Falcón M.S., Simal-Gándara J., Rodrigues A.S., Almeida D.P.F. (2010). Identification and Quantification of Flavonoids in Traditional Cultivars of Red and White Onions at Harvest. J. Food Compos. Anal..

[12] Sellappan S., Akoh C.C. (2002). Flavonoids and Antioxidant Capacity of Georgia-Grown Vidalia Onions. J. Agric. Food Chem..

[13] Bahadoran Z., Mirmiran P., Azizi F. (2013). Dietary Polyphenols as Potential Nutraceuticals in Management of Diabetes: A Review. J. Diabetes Metab. Disord..

[14] Martinez-Gonzalez A.I., Díaz-Sánchez G., de la Rosa L.A., Bustos-Jaimes I., Alvarez-Parrilla E. (2019). Inhibition of α-Amylase by Flavonoids: Structure Activity Relationship (SAR). Spectrochim. Acta A Mol. Biomol. Spectrosc..

[15] Borah M.S., Tiwari A., Sridhar K., Narsaiah K., Nayak P.K., Stephen Inbaraj B. (2023). Recent Trends in Valorization of Food Industry Waste and By-Products: Encapsulation and In Vitro Release of Bioactive Compounds. Foods.

[16] Prokopov T., Chonova V., Slavov A., Dessev T., Dimitrov N., Petkova N. (2018). Effects on the Quality and Health-Enhancing Properties of Industrial Onion Waste Powder on Bread. J. Food Sci. Technol..

[17] Sagar N.A., Pareek S. (2020). Dough Rheology, Antioxidants, Textural, Physicochemical Characteristics, and Sensory Quality of Pizza Base Enriched with Onion (*Allium cepa* L.) Skin Powder. Sci. Rep..

[18] Imeneo V., Piscopo A., Santacaterina S., De Bruno A., Poiana M. (2023). Sustainable Recovery of Antioxidant Compounds from Rossa Di Tropea Onion Waste and Application as Ingredient for White Bread Production. Sustainability.

[19] Bedrníček J., Kadlec J., Laknerová I., Mráz J., Samková E., Petrášková E., Hasoňová L., Vácha F., Kron V., Smetana P. (2020). Onion Peel Powder as an Antioxidant-Rich Material for Sausages Prepared from Mechanically Separated Fish Meat. Antioxidants.

[20] Ucak İ., Khalily R., Abuibaid A.K.M., Ogunkalu O.A. (2018). Maintaining the Quality of Rainbow Trout (*Oncorhynchus mykiss*) Fillets by Treatment of Red Onion Peel Extract during Refrigerated Storage: Assessment of Onion Peel Extract in Fish Quality. Prog. Nutr..

[21] Michalak-Majewska M., Teterycz D., Muszyński S., Radzki W., Sykut-Domańska E. (2020). Influence of Onion Skin Powder on Nutritional and Quality Attributes of Wheat Pasta. PLoS ONE.

[22] Marcillo-Parra V., Tupuna-Yerovi D.S., González Z., Ruales J. (2021). Encapsulation of Bioactive Compounds from Fruit and Vegetable By-Products for Food Application–A Review. Trends Food Sci. Technol..

[23] Buljeta I., Pichler A., Šimunović J., Kopjar M. (2022). Polysaccharides as Carriers of Polyphenols: Comparison of Freeze-Drying and Spray-Drying as Encapsulation Techniques. Molecules.

[24] Stoica F., Condurache N.N., Horincar G., Constantin O.E., Turturică M., Stănciuc N., Aprodu I., Croitoru C., Râpeanu G. (2022). Value-Added Crackers Enriched with Red Onion Skin Anthocyanins Entrapped in Different Combinations of Wall Materials. Antioxidants.

[25] Stoica F., Condurache N.N., Aprodu I., Andronoiu D.G., Enachi E., Stănciuc N., Bahrim G.E., Croitoru C., Râpeanu G. (2022). Value-Added Salad Dressing Enriched with Red Onion Skin Anthocyanins Entrapped in Different Biopolymers. Food Chem. X.

[26] Milea Ș.A., Aprodu I., Enachi E., Barbu V., Râpeanu G., Bahrim G.E., Stănciuc N. (2021). Whey Protein Isolate-Xylose Maillard-Based Conjugates with Tailored Microencapsulation Capacity of Flavonoids from Yellow Onions Skins. Antioxidants.

[27] Elsebaie E.M., Essa R.Y. (2018). Microencapsulation of Red Onion Peel Polyphenols Fractions by Freeze Drying Technicality and Its Application in Cake. J. Food Process Preserv..

[28] Cao H., Saroglu O., Karadag A., Diaconeasa Z., Zoccatelli G., Conte-Junior C.A., Gonzalez-Aguilar G.A., Ou J., Bai W., Zamarioli C.M. (2021). Available Technologies on Improving the Stability of Polyphenols in Food Processing. Food Front..

[29] Flamminii F., Di Mattia C.D., Sacchetti G., Neri L., Mastrocola D., Pittia P. (2020). Physical and Sensory Properties of Mayonnaise Enriched with Encapsulated Olive Leaf Phenolic Extracts. Foods.

[30] Tatasciore S., Santarelli V., Neri L., González Ortega R., Faieta M., Di Mattia C.D., Di Michele A., Pittia P. (2023). Freeze-Drying Microencapsulation of Hop Extract: Effect of Carrier Composition on Physical, Techno-Functional, and Stability Properties. Antioxidants.

[31] González-Ortega R., Faieta M., Di Mattia C.D., Valbonetti L., Pittia P. (2020). Microencapsulation of Olive Leaf Extract by Freeze-Drying: Effect of Carrier Composition on Process Efficiency and Technological Properties of the Powders. J. Food Eng..

[32] Saénz C., Tapia S., Chávez J., Robert P. (2009). Microencapsulation by Spray Drying of Bioactive Compounds from Cactus Pear (*Opuntia ficus-indica*). Food Chem..

[33] Robert P., Torres V., García P., Vergara C., Sáenz C. (2015). The Encapsulation of Purple Cactus Pear (*Opuntia ficus-indica*) Pulp by Using Polysaccharide-Proteins as Encapsulating Agents. LWT-Food Sci. Technol..

[34] Pieczykolan E., Kurek M.A. (2019). Use of Guar Gum, Gum Arabic, Pectin, Beta-Glucan and Inulin for Microencapsulation of Anthocyanins from Chokeberry. Int. J. Biol. Macromol..

[35] Nguyen Q.D., Dang T.T., Nguyen T.V.L., Nguyen T.T.D., Nguyen N.N. (2022). Microencapsulation of Roselle (*Hibiscus sabdariffa* L.) Anthocyanins: Effects of Different Carriers on Selected Physicochemical Properties and Antioxidant Activities of Spray-Dried and Freeze-Dried Powder. Int. J. Food Prop..

[36] Milea Ș.A., Vasile M.A., Crăciunescu O., Prelipcean A.M., Bahrim G.E., Râpeanu G., Oancea A., Stănciuc N. (2020). Co-Microencapsulation of Flavonoids from Yellow Onion Skins and Lactic Acid Bacteria Lead to Multifunctional Ingredient for Nutraceutical and Pharmaceutics Applications. Pharmaceutics.

[37] Nunes G.L., Etchepare M.d.A., Cichoski A.J., Zepka L.Q., Jacob Lopes E., Barin J.S., Flores É.M.d.M., da Silva C.d.B., de Menezes C.R. (2018). Inulin, Hi-Maize, and Trehalose as Thermal Protectants for Increasing Viability of Lactobacillus Acidophilus Encapsulated by Spray Drying. LWT.

[38] Fernandes R.V.D.B., Borges S.V., Botrel D.A. (2014). Gum Arabic/Starch/Maltodextrin/Inulin as Wall Materials on the Microencapsulation of Rosemary Essential Oil. Carbohydr. Polym..

[39] Fernandes R.V.d.B., Silva E.K., Borges S.V., de Oliveira C.R., Yoshida M.I., da Silva Y.F., do Carmo E.L., Azevedo V.M., Botrel D.A. (2017). Proposing Novel Encapsulating Matrices for Spray-Dried Ginger Essential Oil from the Whey Protein Isolate-Inulin/Maltodextrin Blends. Food Bioprocess Technol..

[40] Silva C.R.d.P., Figueiredo J.d.A., Campelo P.H., Silveira P.G., Souza F.d.C.D.A., Yoshida M.I., Borges S.V. (2025). Spray Drying of Aqueous South American Sapote (*Matisia cordata*) Extract: Influence of Dextrose Equivalent and Dietary Fiber on Physicochemical Properties. Food Bioprocess Technol..

[41] Teo A., Lam Y., Lee S.J., Goh K.K.T. (2021). Spray Drying of Whey Protein Stabilized Nanoemulsions Containing Different Wall Materials–Maltodextrin or Trehalose. LWT.

[42] Millinia B.L., Mashithah D., Nawatila R., Kartini K. (2024). Microencapsulation of Roselle (*Hibiscus sabdariffa* L.) Anthocyanins: Effects of Maltodextrin and Trehalose Matrix on Selected Physicochemical Properties and Antioxidant Activities of Spray-Dried Powder. Future Foods.

[43] Jurmanović S., Jug M., Safner T., Radić K., Domijan A.M., Pedisić S., Šimić S., Jablan J., Čepo D.V. (2019). Utilization of Olive Pomace as a Source of Polyphenols: Optimization of Microwave-Assisted Extraction and Characterization of Spray-Dried Extract. J. Food Nutr. Res..

[44] Aliakbarian B., Paini M., Casazza A.A., Perego P. (2015). Effect of Encapsulating Agent on Physical-Chemical Characteristics of Olive Pomace Polyphenols-Rich Extracts. Chem. Eng. Trans..

[45] Waterhouse G.I.N., Sun-Waterhouse D., Su G., Zhao H., Zhao M. (2017). Spray-Drying of Antioxidant-Rich Blueberry Waste Extracts; Interplay Between Waste Pretreatments and Spray-Drying Process. Food Bioprocess Technol..

[46] Zanoni F., Primiterra M., Angeli N., Zoccatelli G. (2020). Microencapsulation by Spray-Drying of Polyphenols Extracted from Red Chicory and Red Cabbage: Effects on Stability and Color Properties. Food Chem..

[47] Edrisi Sormoli M., Langrish T.A.G. (2016). The Use of a Plug-Flow Model for Scaling-up of Spray Drying Bioactive Orange Peel Extracts. Innov. Food Sci. Emerg. Technol..

[48] Lourenço S.C., Moldão-Martins M., Alves V.D. (2020). Microencapsulation of Pineapple Peel Extract by Spray Drying Using Maltodextrin, Inulin, and Arabic Gum as Wall Matrices. Foods.

[49] Paini M., Aliakbarian B., Casazza A.A., Lagazzo A., Botter R., Perego P. (2015). Microencapsulation of Phenolic Compounds from Olive Pomace Using Spray Drying: A Study of Operative Parameters. LWT-Food Sci. Technol..

[50] Santarelli V., Neri L., Sacchetti G., Di Mattia C.D., Mastrocola D., Pittia P. (2020). Response of Organic and Conventional Apples to Freezing and Freezing Pre-Treatments: Focus on Polyphenols Content and Antioxidant Activity. Food Chem..

[51] Robert P., Gorena T., Romero N., Sepulveda E., Chavez J., Saenz C. (2010). Encapsulation of Polyphenols and Anthocyanins from Pomegranate (*Punica granatum*) by Spray Drying. Int. J. Food Sci. Technol..

[52] Benzie I.F.F., Strain J.J. (1999). [2] Ferric Reducing/Antioxidant Power Assay: Direct Measure of Total Antioxidant Activity of Biological Fluids and Modified Version for Simultaneous Measurement of Total Antioxidant Power and Ascorbic Acid Concentration. Methods Enzymol..

[53] Flamminii F., Paciulli M., Di Michele A., Littardi P., Carini E., Chiavaro E., Pittia P., Di Mattia C.D. (2021). Alginate-Based Microparticles Structured with Different Biopolymers and Enriched with a Phenolic-Rich Olive Leaves Extract: A Physico-Chemical Characterization. Curr. Res. Food Sci..

[54] Wu S., Miao S. (2024). Physical Properties and Stickiness of Spray-Dried Food Powders. Spray Drying for the Food Industry: Unit Operations and Processing Equipment in the Food Industry.

[55] Cai Y.Z., Corke H. (2000). Production and Properties of Spray-Dried Amaranthus Betacyanin Pigments. J. Food Sci..

[56] Goula A.M., Adamopoulos K.G. (2010). A New Technique for Spray Drying Orange Juice Concentrate. Innov. Food Sci. Emerg. Technol..

[57] Rodríguez-Hernández G.R., González-García R., Grajales-Lagunes A., Ruiz-Cabrera M.A., Abud-Archila M. (2005). Spray-Drying of Cactus Pear Juice (*Opuntia streptacantha*): Effect on the Physicochemical Properties of Powder and Reconstituted Product. Dry. Technol..

[58] Bassani A., Carullo D., Rossi F., Fiorentini C., Garrido G.D., Reklaitis G.V.R., Bonadies I., Spigno G. (2022). Modeling of a Spray-Drying Process for the Encapsulation of High-Added Value Extracts from Food by-Products. Comput. Chem. Eng..

[59] Quek S.Y., Chok N.K., Swedlund P. (2007). The Physicochemical Properties of Spray-Dried Watermelon Powders. Chem. Eng. Process. Process Intensif..

[60] Tkacz K., Wojdyło A., Michalska-Ciechanowska A., Turkiewicz I.P., Lech K., Nowicka P. (2020). Influence Carrier Agents, Drying Methods, Storage Time on Physico-Chemical Properties and Bioactive Potential of Encapsulated Sea Buckthorn Juice Powders. Molecules.

[61] Dobroslavić E., Elez Garofulić I., Zorić Z., Pedisić S., Roje M., Dragović-Uzelac V. (2023). Physicochemical Properties, Antioxidant Capacity, and Bioavailability of *Laurus nobilis* L. Leaf Polyphenolic Extracts Microencapsulated by Spray Drying. Foods.

[62] Finney J., Buffo R., Reineccius G.A. (2002). Effects of Type of Atomization and Processing Temperatures on the Physical Properties and Stability of Spray-Dried Flavors. J. Food Sci..

[63] Quispe-Condori S., Saldaña M.D.A., Temelli F. (2011). Microencapsulation of Flax Oil with Zein Using Spray and Freeze Drying. LWT-Food Sci. Technol..

[64] Hadree J., Shahidi F., Mohebbi M., Abbaspour M. (2023). Evaluation of Effects of Spray Drying Conditions on Physicochemical Properties of Pomegranate Juice Powder Enriched with Pomegranate Peel Phenolic Compounds: Modeling and Optimization by RSM. Foods.

[65] Landillon V., Cassan D., Morel M.H., Cuq B. (2008). Flowability, Cohesive, and Granulation Properties of Wheat Powders. J. Food Eng..

[66] Leturia M., Benali M., Lagarde S., Ronga I., Saleh K. (2014). Characterization of Flow Properties of Cohesive Powders: A Comparative Study of Traditional and New Testing Methods. Powder Technol..

[67] Rocha J.d.C.G., de Barros F.A.R., Perrone Í.T., Viana K.W.C., Tavares G.M., Stephani R., Stringheta P.C. (2019). Microencapsulation by Atomization of the Mixture of Phenolic Extracts. Powder Technol..

[68] Hong L., Chen L., Ladika M., Li Y., Kim-Habermehl L., Bergman R. (2014). Impact of Particle Size and Surface Charge Density on Redispersibility of Spray-Dried Powders. Colloids Surf. A Physicochem. Eng. Asp..

[69] Ozkan G., Franco P., De Marco I., Xiao J., Capanoglu E. (2019). A Review of Microencapsulation Methods for Food Antioxidants: Principles, Advantages, Drawbacks and Applications. Food Chem..

[70] Mensink M.A., Frijlink H.W., Van Der Voort Maarschalk K., Hinrichs W.L.J. (2015). Inulin, a Flexible Oligosaccharide I: Review of Its Physicochemical Characteristics. Carbohydr. Polym..

[71] Lacerda E.C.Q., Calado V.M.D.A., Monteiro M., Finotelli P.V., Torres A.G., Perrone D. (2016). Starch, Inulin and Maltodextrin as Encapsulating Agents Affect the Quality and Stability of Jussara Pulp Microparticles. Carbohydr. Polym..

[72] Castro-Muñoz R., Barragán-Huerta B.E., Yáñez-Fernández J. (2015). Use of Gelatin-Maltodextrin Composite as an Encapsulation Support for Clarified Juice from Purple Cactus Pear (*Opuntia stricta*). LWT-Food Sci. Technol..

[73] Rashid S., Rakha A., Butt M.S., Asgher M. (2018). Physicochemical and Techno-Functional Characterization of Inulin Extracted from Chicory Roots and Jerusalem Artichoke Tubers and Exploring Their Ability to Replace the Fat in Cakes. Prog. Nutr..

[74] Cui S.W., Nie S., Roberts K.T. (2011). Functional Properties of Dietary Fiber.

[75] Walz M., Hirth T., Weber A. (2018). Investigation of Chemically Modified Inulin as Encapsulation Material for Pharmaceutical Substances by Spray-Drying. Colloids Surf. A Physicochem. Eng. Asp..

[76] Mousavi Kalajahi S.E., Ghandiha S. (2022). Optimization of Spray Drying Parameters for Encapsulation of Nettle (*Urtica dioica* L.) Extract. LWT.

[77] Mahmoudi L., Tavakoilpour H., Roozbeh-Nasiraie L., Kalbasi-Ashtari A. (2020). Ultrasonication and Encapsulation of Butcher Broom (*Ruscus hyrcanus* L.) Extract and Its Bioactive Effects on Qualitative Properties, Oxidative Stability and Shelf Life of Cake. Sustain. Chem. Pharm..

[78] Faieta M., Corradini M.G., Di Michele A., Ludescher R.D., Pittia P. (2020). Effect of Encapsulation Process on Technological Functionality and Stability of Spirulina Platensis Extract. Food Biophys..

[79] Papoutsis K., Golding J.B., Vuong Q., Pristijono P., Stathopoulos C.E., Scarlett C.J., Bowyer M. (2018). Encapsulation of Citrus By-Product Extracts by Spray-Drying and Freeze-Drying Using Combinations of Maltodextrin with Soybean Protein and ι-Carrageenan. Foods.

[80] Zhang J., Zhang C., Chen X., Quek S.Y. (2020). Effect of Spray Drying on Phenolic Compounds of Cranberry Juice and Their Stability during Storage. J. Food Eng..

[81] Bernstein A., Noreña C.P.Z. (2015). Encapsulation of Red Cabbage (*Brassica oleracea* L. var. capitata L. f. rubra) Anthocyanins by Spray Drying Using Different Encapsulating Agents. Braz. Arch. Biol. Technol..

[82] Castel V., Rubiolo A.C., Carrara C.R. (2018). Brea Gum as Wall Material in the Microencapsulation of Corn Oil by Spray Drying: Effect of Inulin Addition. Food Res. Int..

[83] Carneiro H.C.F., Tonon R.V., Grosso C.R.F., Hubinger M.D. (2013). Encapsulation Efficiency and Oxidative Stability of Flaxseed Oil Microencapsulated by Spray Drying Using Different Combinations of Wall Materials. J. Food Eng..

[84] Flamminii F., Di Mattia C.D., Nardella M., Chiarini M., Valbonetti L., Neri L., Difonzo G., Pittia P. (2020). Structuring Alginate Beads with Different Biopolymers for the Development of Functional Ingredients Loaded with Olive Leaves Phenolic Extract. Food Hydrocoll..

[85] Kacuráková M., Capek P., Sasinková V., Wellner N., Ebringerová A. (2000). FT-IR Study of Plant Cell Wall Model Compounds: Pectic Polysaccharides and Hemicelluloses. Carbohydr. Polym..

[86] Krysa M., Szymańska-Chargot M., Zdunek A. (2022). FT-IR and FT-Raman Fingerprints of Flavonoids—A Review. Food Chem..

[87] Cervantes-Martínez C.V., Medina-Torres L., González-Laredo R.F., Calderas F., Sánchez-Olivares G., Herrera-Valencia E.E., Gallegos Infante J.A., Rocha-Guzman N.E., Rodríguez-Ramírez J. (2014). Study of Spray Drying of the Aloe Vera Mucilage (*Aloe vera barbadensis* Miller) as a Function of Its Rheological Properties. LWT-Food Sci. Technol..

[88] Medina-Torres L., GarcÍa-Cruz E.E., Calderas F., González Laredo R.F., Sánchez-Olivares G., Gallegos-Infante J.A., Rocha-Guzmán N.E., RodrÍguez-RamÍrez J. (2013). Microencapsulation by Spray Drying of Gallic Acid with Nopal Mucilage (*Opuntia ficus indica*). LWT-Food Sci. Technol..

[89] Barrett D.M., Somogyi L., Ramaswamy H.S. (2004). Processing Fruits: Science and Technology.

[90] Gharsallaoui A., Roudaut G., Chambin O., Voilley A., Saurel R. (2007). Applications of Spray-Drying in Microencapsulation of Food Ingredients: An Overview. Food Res. Int..

[91] Chen Y., Martynenko A. (2017). Storage Stability of Cranberry Puree Products Processed with Hydrothermodynamic (HTD) Technology. LWT-Food Sci. Technol..

[92] Saikia S., Mahnot N.K., Mahanta C.L. (2015). Effect of Spray Drying of Four Fruit Juices on Physicochemical, Phytochemical and Antioxidant Properties. J. Food Process. Preserv..

[93] Robert P., García P., Reyes N., Chávez J., Santos J. (2012). Acetylated Starch and Inulin as Encapsulating Agents of Gallic Acid and Their Release Behaviour in a Hydrophilic System. Food Chem..

[94] Singh N., Chawla D., Singh J. (2004). Influence of Acetic Anhydride on Physicochemical, Morphological and Thermal Properties of Corn and Potato Starch. Food Chem..

[95] Stevens C.V., Meriggi A., Booten K. (2001). Chemical Modification of Inulin, a Valuable Renewable Resource, and Its Industrial Applications. Biomacromolecules.

[96] Shahidi F., Naczk M. (2003). Phenolics in Food and Nutraceuticals.

[97] Ding Z., Tao T., Wang X., Prakash S., Zhao Y., Han J., Wang Z. (2020). Influences of Different Carbohydrates as Wall Material on Powder Characteristics, Encapsulation Efficiency, Stability and Degradation Kinetics of Microencapsulated Lutein by Spray Drying. Int. J. Food Sci. Technol..

[98] Chandra A., Nair M.G., Iezzoni A.F. (1993). Isolation and Stabilization of Anthocyanins from Tart Cherries (*Prunus cerasus* L.). J. Agric. Food Chem..

[99] Zhang Y., Zhang C., Xu C., Deng Y., Wen B., Xie P., Huang L. (2022). Effect of Geographical Location and Soil Fertility on Main Phenolic Compounds and Fatty Acids Compositions of Virgin Olive Oil from Leccino Cultivar in China. Food Res. Int..

[100] Flores F.P., Singh R.K., Kong F. (2014). Physical and Storage Properties of Spray-Dried Blueberry Pomace Extract with Whey Protein Isolate as Wall Material. J. Food Eng..

[101] Tsali A., Goula A.M. (2018). Valorization of Grape Pomace: Encapsulation and Storage Stability of Its Phenolic Extract. Powder Technol..

[102] Pokorný J., Schmidt Š. (2003). The Impact of Food Processing in Phytochemicals: The Case of Antioxidants. Phytochemical Functional Foods.

[103] Zhu L., Du Q., Shi T., Huang J., Deng J., Li H., Cai F., Chen Q. (2024). Analysis of Total Flavonoid Variation and Other Functional Substances in RILs of Tartary Buckwheat, with Near-Infrared Model Construction for Rapid Non-Destructive Detection. Agronomy.

[104] Fracassetti D., Del Bo’ C., Simonetti P., Gardana C., Klimis-Zacas D., Ciappellano S. (2013). Effect of Time and Storage Temperature on Anthocyanin Decay and Antioxidant Activity in Wild Blueberry (*Vaccinium angustifolium*) Powder. J. Agric. Food Chem..

[105] Zorić Z., Dragović-Uzelac V., Pedisić S., Kurtanjek Ž., Elez Garofulić I. (2014). Kinetics of the Degradation of Anthocyanins, Phenolic Acids and Flavonols During Heat Treatments of Freeze-Dried Sour Cherry Marasca Paste. Food Technol. Biotechnol..

[106] Zorić Z., Pelaić Z., Pedisić S., Elez Garofulić I., Bursać Kovačević D., Dragović–Uzelac V. (2017). Effect of Storage Conditions on Phenolic Content and Antioxidant Capacity of Spray Dried Sour Cherry Powder. LWT-Food Sci. Technol..

[107] Shakoor A., Zhang C., Xie J., Yang X. (2022). Maillard Reaction Chemistry in Formation of Critical Intermediates and Flavour Compounds and Their Antioxidant Properties. Food Chem..

